# Microfluidic Capture of Mycobacterium tuberculosis from Clinical Samples for Culture-Free Whole-Genome Sequencing

**DOI:** 10.1128/spectrum.01114-23

**Published:** 2023-06-26

**Authors:** Nabila Ismail, Anzaan Dippenaar, George Morgan, Melanie Grobbelaar, Felicia Wells, Jessica Caffry, Cristiana Morais, Krzysztof Gizynski, David McGurk, Eduardo Boada, Heather Murton, Robin M. Warren, Annelies Van Rie

**Affiliations:** a Division of Molecular Biology and Human Genetics, South African Medical Research Council Centre for Tuberculosis Research, DSI-NRF Centre of Excellence for Biomedical Tuberculosis Research, Faculty of Medicine and Health Sciences, Stellenbosch University, Cape Town, South Africa; b Tuberculosis Omics Research Consortium, Family Medicine and Population Health, Institute of Global Health, Faculty of Medicine and Health Sciences, University of Antwerp, Antwerp, Belgium; c QuantuMDx Ltd., Newcastle upon Tyne, United Kingdom; University of Pretoria

**Keywords:** *Mycobacterium tuberculosis*, tuberculosis, whole-genome sequencing, microfluidics, culture-free sequencing, cell capture

## Abstract

Mycobacterium tuberculosis whole-genome sequencing (WGS) is a powerful tool as it can provide data on population diversity, drug resistance, disease transmission, and mixed infections. Successful WGS is still reliant on high concentrations of DNA obtained through M. tuberculosis culture. Microfluidics technology plays a valuable role in single-cell research but has not yet been assessed as a bacterial enrichment strategy for culture-free WGS of M. tuberculosis. In a proof-of-principle study, we evaluated the use of Capture-XT, a microfluidic lab-on-chip cleanup and pathogen concentration platform to enrich M. tuberculosis bacilli from clinical sputum specimens for downstream DNA extraction and WGS. Three of the four (75%) samples processed by the microfluidics application passed the library preparation quality control, compared to only one of the four (25%) samples not enriched by the microfluidics M. tuberculosis capture application. WGS data were of sufficient quality, with mapping depth of ≥25× and 9 to 27% of reads mapping to the reference genome. These results suggest that microfluidics-based M. tuberculosis cell capture might be a promising method for M. tuberculosis enrichment in clinical sputum samples, which could facilitate culture-free M. tuberculosis WGS.

**IMPORTANCE** Diagnosis of tuberculosis is effective using molecular methods; however, a comprehensive characterization of the resistance profile of Mycobacterium tuberculosis often requires culturing and phenotypic drug susceptibility testing or culturing followed by whole-genome sequencing (WGS). The phenotypic route can take anywhere from 1 to >3 months to result, by which point the patient may have acquired additional drug resistance. The WGS route is a very attractive option; however, culturing is the rate-limiting step. In this original article, we provide proof-of-principle evidence that microfluidics-based cell capture can be used on high-bacillary-load clinical samples for culture-free WGS.

## INTRODUCTION

Since the characterization of the complete genome of Mycobacterium tuberculosis in 1998 (1), molecular biology has increasingly played a role in tuberculosis (TB) research and care. Recently, there is an increasing interest in the use of next-generation technologies, such as targeted deep sequencing and whole-genome sequencing (WGS). Targeted deep sequencing could play a role in the diagnosis of drug resistance by identifying variants in candidate resistance genes directly in sputum samples, but current assays have relatively poor performance when the sputum bacillary load is low (2). WGS has applications beyond the diagnosis of drug resistance. Since 2009, WGS has contributed to our understanding of M. tuberculosis transmission (3), mixed infections (4), the distinction between relapse and reinfection (5), mechanisms of drug resistance (6, 7), genetic M. tuberculosis diversity at population level (8, 9), and within-host mycobacterial diversity (10–12). Currently, M. tuberculosis culture is required to obtain sufficient DNA for WGS. The culture step can reduce the true M. tuberculosis strain diversity present in the sputum sample, which may result in the elimination of minor populations, drug-tolerant populations, or persister subpopulations (13).

To date, most M. tuberculosis WGS has been performed on DNA extracted from purified subcultures. In 2015, pretreatment steps for human DNA removal and bead cleanup for enrichment of M. tuberculosis DNA allowed successful WGS of primary early-positive liquid cultures (14). Culture-free WGS has remained challenging as sputum is a viscous substance comprised of human cells and a plethora of microbial cells from the complex oral and lung microbiome, with often low M. tuberculosis representation. Brown et al. were the first to succeed in culture-free WGS employing RNA baits to selectively capture M. tuberculosis DNA (SureSelect; Agilent) (15), a method subsequently adopted by several other studies (13, 16–18). In 2020, Goig et al. were the first to directly sequence smear-negative sputum samples but, however, with a relatively low (55%) success rate (18). The six published culture-free M. tuberculosis WGS studies to date present results only on 138 predominantly smear-positive sputum samples. The varying success in obtaining sequencing data in these studies highlights the challenge of producing sequence-able M. tuberculosis DNA directly from a sputum sample.

The application of a technology that selectively enriches M. tuberculosis directly from sputum for DNA extraction could potentially increase sequence-able M. tuberculosis DNA without introducing culture bias. In this proof-of-concept study, we assessed the performance of Capture-XT, a microfluidic lab-on-chip cleanup and pathogen concentration technology, as a potential front end for downstream culture-free M. tuberculosis WGS of clinical sputum samples.

## RESULTS

The amount of DNA extracted ranged from 0.033 to 0.205 ng/μL for the four BD MycoPrep-treated samples and ranged from 0.016 to 0.464 ng/μL for the four samples processed with the QuantuMDx thinning reagent (Table 1). Creation of libraries for WGS succeeded in three of the four samples that underwent microfluidic M. tuberculosis capture compared to one of the four samples that was not run through the microfluidics device. Samples that underwent bacterial capture had higher library concentrations (4.6, 10.5, and 41 nM) compared to the sample that was not run through the device (Table 1).

**TABLE 1:** Comparison of library preparation and WGS data quality metrics^*c*^

Sample no.	Bacterial load (Xpert *C_T_* value)	Liquefaction method	Microfluidic capture	DNA concn^*b*^ (ng/μL)	Avg library fragment size (bp)	Library concn^*a*^ (nM)	Library prepn QC status	WGS data
1	High (14)	BD MycoPrep	Yes	0.033			Failed	
2	High (14)	BD MycoPrep	Yes	0.120	595	41.0	Passed	59× DoC; ±27% mapped reads
3	High (14)	BD MycoPrep	No	0.186			Failed	
4	High (14)	BD MycoPrep	No	0.205	601	1.4	Passed	Run failed
5	High (14)	QuantuMDx	Yes	0.042	465	10.5	Passed	25× DoC; ±9% mapped reads
6	High (14)	QuantuMDx	Yes	0.042	400	4.6	Passed	Run failed
7	High (14)	QuantuMDx	No	0.464			Failed	
8	High (14)	QuantuMDx	No	0.016			Failed	

a Library concentration calculated using the formula: $\frac{\text{library concentration in}\;\frac{\text{ng}}{\text{μl}}\;\text{×}\;{\text{10}}^{\text{6}}}{\text{660}\;\frac{\text{g}}{\text{mol}}\;\text{×}\;\text{average fragment size}}$ with average fragment size data taken from the LabChip profile.

b Average concentration derived from *rpoB* quantitative PCR done in triplicate.

c DoC, depth of coverage; QC, quality control; *C_T_*, cycle threshold.

Four samples passed the WGS quality control requirements of more than 1 ng/μL and fragment size between 350 and 650 bp: sample 2 (BD MycoPrep treated, captured), sample 4 (BD MycoPrep treated, noncaptured), sample 5 (QuantuMDx treated, captured), and sample 6 (QuantuMDx treated, noncaptured) (Table 1). Due to a technical error, the sequencing run with samples 4 and 6 failed, and no WGS data could be produced. Sample 2 (BD MycoPrep treated, captured) had a depth of coverage of 59× with ±27% of the total number of reads mapping to the H37Rv reference genome. Sample 5 (QuantuMDx treated, captured) had a depth of coverage of 25×, and ±9% of reads mapped to the reference genome.

## DISCUSSION

WGS of M. tuberculosis has already made invaluable contributions to tuberculosis research, but the culture step needed to obtain sufficient amounts of DNA limits its applications (19). Currently, most WGS methods are developed for sequencing on a “purified” M. tuberculosis subculture or on a primary liquid culture shortly after flagging positive for mycobacterial growth (14). WGS using DNA obtained from specimens without a culture enrichment step could speed up the turnaround time from sample collection to sequence results, which is essential for patient care. This would also allow the elucidation of the true population diversity that is otherwise biased by the culture process (20, 21). Bait capturing of M. tuberculosis DNA for culture-free WGS has not been consistently successful and adds significant costs and complexity to the sample preparation (13, 15, 17). Similarly, culture-free targeted deep sequencing methods have had limited and inconsistent success in processing smear-negative specimens (2).

Microfluidics applications to separate bacterial cells in unprocessed samples already play a valuable role in single-cell research (22). Certain microfluidic applications have incorporated cultivation and visual estimation of growth, but this is possible only for rapidly growing bacteria (23, 24). For clinical sputum samples, microfluidic applications could capture the mycobacteria and reduce the amounts of nonmycobacteria, thus enriching the M. tuberculosis for downstream analyses. To date, only one study has evaluated the use of a microfluidic sample preparation for M. tuberculosis enrichment. This study found that a low input of M. tuberculosis (~10,000 cells) resulted in efficient cell concentration, lysis, and purification for downstream enrichment PCR and barcoding for whole-genome shotgun sequencing (25). This study was, however, not performed directly on a sputum sample but used aliquots of two clinical M. tuberculosis culture isolates (25).

Our study, the first assessing the use of microfluidic pathogen-concentration technology for M. tuberculosis sequencing directly from sputum, provides proof-of-principle evidence that microfluidics-based cell capture can be used for culture-free WGS. Several limitations should be considered when interpreting the results. First, the sample size of this pilot study was small, including only 8 experiments. Furthermore, one of the two sequencing runs failed and could not be repeated as all material had been used. Second, we pooled sputa for comparability purposes. The mycobacterial load of the pooled sample tested was high, with an Xpert MTB/RIF Ultra cycle threshold (*C_T_*) value of 14 relating to a smear-positive sample with a smear grade of 2+ or 3+ (26). Further studies are thus needed to evaluate the performance of microfluidics capture on clinical sputum samples with various mycobacterial loads.

In conclusion, despite the progress in sequencing technology and bioinformatics analyses, limited progress has been made in methods to prepare clinical samples for M. tuberculosis WGS directly from sputum. While our data show promising results, larger studies are needed to evaluate the use of microfluidic pathogen-concentration techniques for direct sequencing of clinical sputum samples.

## MATERIALS AND METHODS

### Preparation of sputum samples.

Sputum samples (*n* = 13) from patients residing in the Cape Town metropolitan area under evaluation for TB (University of Cape Town Human Research Ethics Committee protocol number 546/2018) were pooled, mixed using a vortex mixer to achieve sample homogeneity, and split into eight 1.5-mL aliquots. The pooled clinical sputum sample had a high mycobacterial load with an Xpert MTB/RIF Ultra (assay version 3) *C_T_* value of 14 (performed on the GeneXpert II system), corresponding to about 10^7^ CFU/mL (27). Pooling was necessary to create a homogeneous sample with a high mycobacterial load.

### Sputum sample liquefaction.

Two pretreatment methods were used. Four samples were processed by the standard *N*-acetyl-l-cysteine (NALC)–NaOH method which has both liquefaction and decontamination properties. Sputum pretreated with the NALC-NaOH BBL MycoPrep (Becton, Dickinson, NJ, USA) solution was incubated at room temperature for 15 min, neutralized with phosphate-buffered saline (PBS) (up to 50 mL), and centrifuged at 3,000 × *g* for 15 min, and the supernatant was removed. The other four samples were processed using the proprietary QuantuMDx thinning reagent, which has only liquefaction properties, before incubation for 1 h. For samples submitted for capture (*n* = 2 for each method), the pellet was then resuspended in 5 mL QuantuMDx GP buffer (10% [vol/vol] glycerol, 0.2% [vol/vol] Pluronic F68, and 0.01 M EDTA).

### Mycobacterial capture.

Four of the eight samples were processed using the QuantuMDx microfluidic capture device, of which two had been pretreated with BD MycoPrep and two with the QuantuMDx thinning reagent. First, samples underwent ion reduction by mixing with ion exchange resin to ensure that the conductivity of the sample was suitable for the dielectrophoresis (DEP) device. Samples were then diluted (1:10) in GP buffer and run through the microfluidic system, which was driven by positive pressure supplied by an FLPG Plus (Fluigent) and regulated using a Flow EZ (Fluigent). Capture of M. tuberculosis cells was performed by applying an alternating current across the electrodes, the sinusoidal waveform was produced using an arbitrary waveform generator (AFG1022; Tektronix), and the signal was amplified (9250; Tabor Electronics). After processing the sample, the current was halted and any captured cells were eluted in 50 μL of GP buffer for downstream processing.

### Lysis, DNA extraction, and DNA quantification.

To extract the DNA, the eight samples were treated overnight with 10 mg/mL lysozyme (Roche, Basel, Switzerland) with agitation at 37°C followed by cetyltrimethylammonium bromide (CTAB) DNA extraction (28). DNA was precipitated using isopropanol overnight, and dried DNA for each sample was resuspended in 12 μL of Tris-EDTA.

Real-time PCR quantification of the extracted DNA was performed in triplicate by amplifying the single-copy *rpoB* gene (forward primer, 5′ ACG GTC GCT TCG TCG AG 3′; reverse primer, 5′ GGG CAC GTA CTC CAC CTC 3′) using a standard curve. Each 10-μL reaction solution comprised nuclease-free water (23.5 μL), Qiagen HotStarTaq Plus master mix (5 μL), SYTO9 (1 μL), SSO Advanced (5 μL) primer mix (0.5 μL), and DNA (1 μL). The amplification protocol consisted of an initial activation step of 95°C for 30 s, followed by 40 cycles of 95°C for 15 s and 60°C for 30 s, with a change of 1.6°C/s increments, and a melting step of 65°C for 15 s and 95°C for 15 s with a change of 0.2°C/s increments. All reactions were performed using QuantStudio 5 (Thermo Fisher Scientific).

### Library preparation and quality control.

The entire volume of DNA left after quantification (~9 μL) was normalized to 30 μL with nuclease-free water. The Nextera DNA Flex library prep kit (Illumina, CA, USA) was used per the manufacturer’s instructions for tagmentation and posttagmentation cleanup. Amplification of tagmented DNA was done using an average of 13 PCR cycles. Library cleanup was performed with diluted sample purification beads (SPB) at a 0.5× bead-to-DNA ratio, followed by a second cleanup with pure SPB. The samples were washed twice using 80% ethanol. Libraries captured on the beads were eluted with an end volume of 30 μL resuspension buffer. Quantification was done with the Qubit double-stranded DNA (dsDNA) high-sensitivity (HS) assay on a Qubit 4 fluorometer (Thermo Fisher Scientific, MA, USA), and the library fragments were analyzed with a high-sensitivity LabChip assay. Thereafter, the library molarity was calculated, and libraries were pooled in equimolar concentrations.

### Whole-genome sequencing and quality assessment of WGS data.

Libraries with a DNA concentration of at least 1 ng/μL and a fragment size between 350 and 650 bp were considered eligible for WGS. The library pool (2 nM) and PhiX control (10 nM) were denatured and diluted with 0.1 M NaOH, 200 mM Tris-HCl, pH 7.0, and hybridization (HT1) buffer to 20 pM. The 20 pM libraries and 20 pM PhiX (3% spike) control were combined to a final volume of 550 μL to a concentration of 1.3 pM. A thawed MiniSeq 300-cycle high-output cartridge and flow cell were loaded with 500 μL of the final 1.3 pM library spiked with 3% PhiX control.

WGS analysis was done using the XBS pipeline (29), which provides a summary of metrics to assess the overall quality of the WGS data including genome-wide depth of coverage and the percentage of reads that mapped to the M. tuberculosis H37Rv reference genome (NC_000962.3).

### Data availability.

Reads are deposited in the European Nucleotide Archive (accession number PRJEB55527).
